# FDG-PET in lymphoma

**DOI:** 10.1186/1470-7330-14-S1-O23

**Published:** 2014-10-09

**Authors:** Michel Meignan

**Affiliations:** 1 LYSA Imaging group, Department of Nuclear Medicine, University Paris Est Créteil, France

## 

FDG-PET has changed lymphoma management at staging and for response assessment due to its capability to improve disease characterization and treatment selection. New recommendations have been made by the ICML working group in 2014. FDG-PET is now recommended for staging in the vast majority of lymphoma since almost all types of lymphoma are FDG-avid. It has been shown recently that FDG-PET due to its high sensitivity for bone marrow involvement in Hodgkin lymphoma can obviate the bone marrow biopsy. In addition FDG PET may be used to target biopsy and based on the local SUVmax to detect transformation.

For response assessment after treatment or during chemotherapy the HIP2007 criteria have now been replaced by the Deauville criteria using a 5-point scale. Initially proposed for the evalaution of interim PET these criteria are now extended for end treatment evaluation. The residual activity observed after treatment is scored against different levels of backgroung i.e: 1.no uptake, 2. uptake uptake ≤ mediastinum but ≤ liver, 3. uptake > mediastinum, 4. uptake moderately higher than liver, 5. uptake markedly higher than liver and/or new lesions. A score 4 has been chosen as the threshold for positivity both at interim and end treatment. The value of this choice has been demonstrated in validation studies. Using these criteria FDG PET has demonstrated its prognostic value early in the course of the treatment or at end treatment in Hodgkin lymphoma, Diffuse large B cell lymphoma and Follicular Lymphoma. A tailored therapy based on interim PET is under study in many ongoing trials but at the present time it is not recommendend to change therapy on the basis of the results of an interim FDG-PET. Quantitative analysis of metabolic volume at staging or Δ SUVmax response assessment and their prognostic values and various trends in the future will be discussed.
